# Slightly perturbing the arm influences choices between multiple targets

**DOI:** 10.3758/s13414-023-02680-x

**Published:** 2023-02-28

**Authors:** Veronica Hadjipanayi, Eli Brenner, Emily M. Crowe

**Affiliations:** 1 School of Psychological Science, University of Bristol, Bristol, UK; 2 ﻿Department of Human Movement Sciences, Faculty of Behavioural and Movement Science, Institute of Brain and Behaviour Amsterdam, Vrije Universiteit Amsterdam, Amsterdam, The Netherlands

**Keywords:** Background motion, Interception task, Fast choices, Perturbations, Posture

## Abstract

We constantly make choices about how to interact with objects in the environment. Do we immediately consider changes in our posture when making such choices? To find out, we examined whether motion in the background, which is known to influence the trajectory of goal-directed hand movements, influences participants’ choices when suddenly faced with two options. The participants’ task was to tap on as many sequentially presented targets as possible within 90 seconds. Sometime after a new target appeared, it split into two targets and participants had to choose which of them to hit. Shortly before the split, the background moved in a way that was expected to result in the finger shifting slightly towards one of the two new targets. We examined whether such shifts influenced the choice between the two targets. The moving background influenced the finger movements in the expected manner: participants moved in the direction of the background motion. It also influenced the choice that participants made between the two targets: participants more frequently chose the target in the direction of the background motion. There was a positive correlation across participants between the magnitude of the response to background motion and the bias to choose the target in the direction of such motion. Thus, people consider sudden changes in their posture when choosing between different movement options.

In our daily life we often have to make quick choices. For example, when we accidentally drop a glass, we quickly have to choose whether to try to catch it before it hits the floor and breaks, or whether to let it fall and step away to reduce the risk of getting hurt. When selecting how to move, people consider the benefits and costs associated with each option (Brenner & Smeets, 2022; De Comite et al., 2022; Eloka & Franz, 2011; Trommershäuser et al., 2003). In particular, when a choice suddenly appears, people consider the options in relation to how their hand is moving at that moment (Brenner & Smeets, 2015b, 2022; Kurtzer et al., 2020). What if the way their hand is moving is perturbed? Will they consider the perturbed movement, or is the choice based on the original plan?

When making goal-directed movements, sudden motion in the background pulls our hand in the direction of motion, and thereby away from the target we were aiming for (Brenner & Smeets, 2015a; Crowe et al., 2021, 2022; Gomi et al., 2006; Saijo et al., 2005). We use this to examine whether the instantaneous, perturbed movement is considered when selecting a target, or whether the choice is based on the planned movement. An advantage of perturbing the movement in this manner rather than mechanically is that it does not involve external forces that the participant might have to consider.

We used a fast-tapping task in which participants were instructed to hit as many targets as possible within 90 seconds. Each trial started with a single target being presented. After 300 ms, this target split into two targets, such that participants had to choose which of the two targets to hit. Just before the choice was presented, the 600 dots that constituted the background moved in a direction orthogonal to the movement towards the target. We expected the participants’ fingers to move slightly in the direction of this background motion. If these adjustments to the movement are considered when making the choice, we expect the background motion to bias the choice towards the target that is in the direction of background motion.

## Method

### Participants

Nineteen right-handed participants (five female; age 29 ± 6 years; mean ± standard deviation) volunteered to take part in the experiment.

### Set-up

The experiment was conducted in a normally illuminated room. The stimuli were back-projected at 120 Hz with a resolution of 800 × 600 pixels onto a 1.25m × 1.0m acrylic rear-projection screen (Techplex 15, Stewart Filmscreen Corporation, Torrance, CA, USA) which was tilted backward by 30°. An Optotrak 3020 camera (Northern Digital) that was placed at about shoulder height to the left of the screen measured the position of a marker (an infrared light-emitting diode) attached to the nail of the index finger of the participant’s dominant finger at 500 Hz. The Optotrak also recorded the position of a second marker attached to the side of the screen in order to synchronize the movement data (marker positions) with the stimulus presentation. This second marker stopped emitting infrared light so that its position was registered as “missing” when a flash was presented at the top left corner of the screen (where a light-sensor was placed to detect the flash).

### Calibration

Before each measurement, the participant positioned their fingertip at four indicated positions on the screen. This was used to relate all further positions of the fingertip to the projected images, automatically correcting for the fact that the marker was attached to the nail rather than the tip of the finger.

### Stimulus and procedure

Participants stood in front of the screen onto which the stimuli were projected. They were free to move as they wanted. Their task was to tap on as many targets as possible within 90 seconds by lifting their finger off the screen and moving it to the chosen target. Each movement to a new target was considered to be a trial. Participants completed a practice block of trials to familiarize themselves with the task, followed by five experimental blocks. Each of the six blocks lasted 90 seconds. The number of trials that participants completed depended on their performance: The quicker they were, the more trials they completed. On average, participants completed 623 trials across the five experimental blocks.

**Fig. 1:**
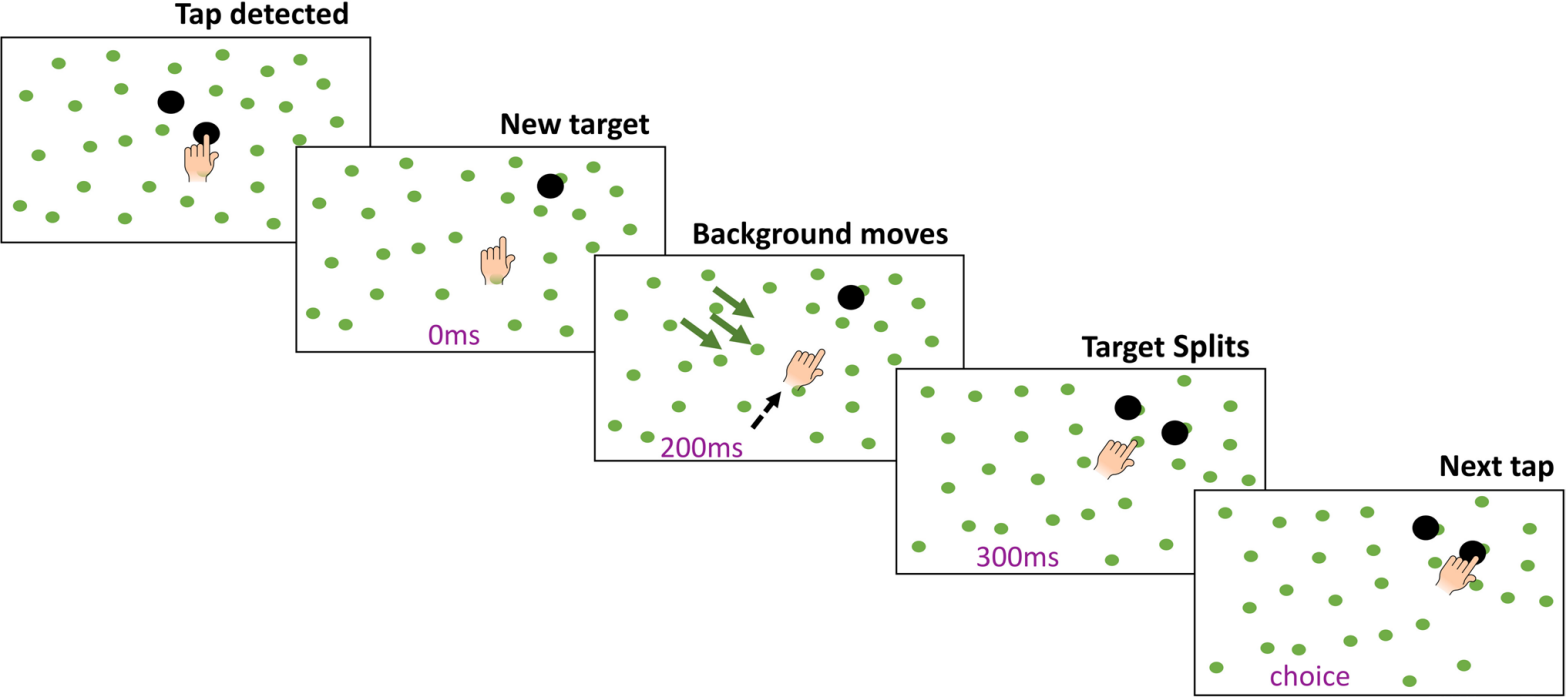
Schematic representation of one trial. The previous trial ended when the finger tapped on the screen. A new target was then presented at a fixed distance from where the finger had tapped. After 200 ms, the background started moving in one of the two directions orthogonal to the direction to the new target. After another 100 ms, the background stopped moving and the target split into two targets that were at equal distances from the initial target position; one in the direction in which the background moved, and the other in the opposite direction. The trial ended when the participant tapped on one of the targets

Each trial started with a single target being presented 177 mm from the position at which the finger last tapped the screen in a direction that was chosen at random from all possible positions that were within 400 mm of the screen centre (Fig. 1). The targets were black discs with a radius of 13.3 mm. There were also 600 small green dots with a radius of 7.4 mm that constituted the background. Exactly 200 ms after the initial target appeared, these background dots started moving at a speed of 102 cm/s. Exactly 100 ms later, 300 ms after the initial target appeared, the background dots stopped moving and the initial target split into two identical targets. The two new targets were at the same 177 mm distance from the position of the previous tap. One was 10° clockwise and the other 10° anti-clockwise from the initial target. Participants had to tap on either of these two targets. In half of the trials, the background moved in the direction of the clockwise target and in the other half in the direction of the anti-clockwise target (always orthogonal to the line connecting the previous tap and the initial target). The trial ended when the participant tapped on the screen. Taps were detected on-line on the basis of the acceleration of the marker (threshold of 50 m/s^2^ orthogonal to the screen) after ensuring that the finger was close to the screen (within 5 mm). If the finger position was within the bounds of one of the two targets when the tap was detected the participant was considered to have hit that target and a sound indicated that the tap was successful. Otherwise, we attributed the tap to the target closest to the finger but since they missed that target no sound was heard (4% of the taps).

### Data analysis

**Fig. 2:**
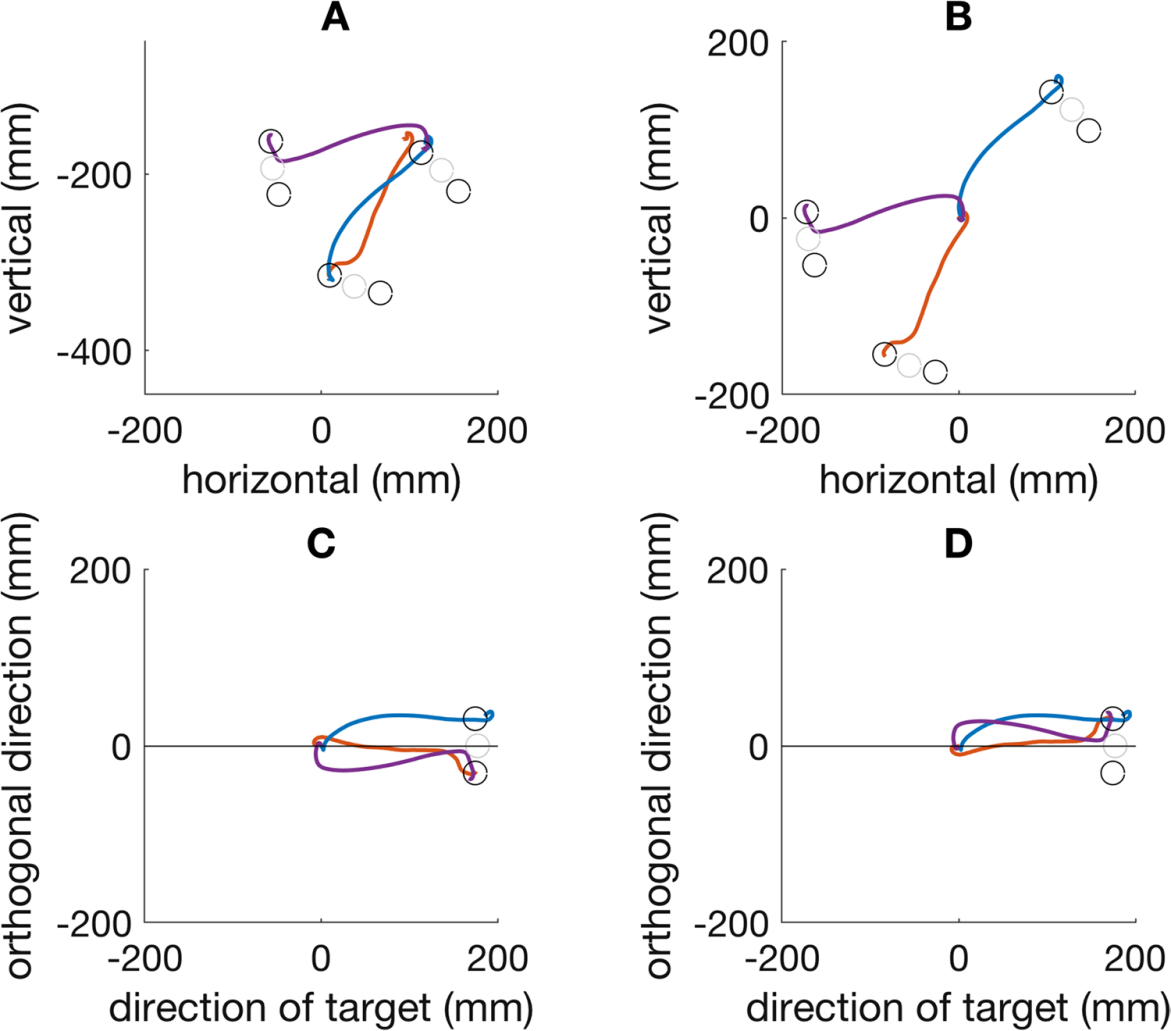
Aligning the trajectories. **A** Finger trajectories on three consecutive trials: red trajectory moving down and to the left, then blue trajectory moving up and to the right, and finally purple trajectory moving to the left. The grey circles are the original target positions. The black circles are the two new positions. **B** All the starting points are shifted to a single position. **C** They are then all rotated around that position such that the targets are to the right. **D** Finally, if the background moved towards the clockwise target (downwards in **C**), the trajectories are reflected across the line through the tap and the initial target (horizontal line in **C** and **D**). The target in the direction of background motion was chosen on all three trials in this example (all three trajectories end in upper target in **D**). (Colour figure online)

Trials in which data was missing after background motion onset or where it took more than 3 standard deviations longer than the mean time taken by the participant in question were excluded from the analysis. These criteria excluded 3.9% of the trials. Since targets appeared in random directions, the movements were also in random directions (Fig. 2A). To be able to compare the responses to background motion and the choices across trials, we first shifted all trajectories such that the movements started at the same position ([0, 0] in Fig. 2B). We then rotated all trajectories around this common starting position such that the positions of the initial target were aligned (at coordinates [0, 177] in Fig. 2C). Finally, in trials in which the background motion moved towards the clockwise target (downwards with respect to the rotated trajectory), the finger trajectories were reflected in the vertical direction (the vertical coordinate was multiplied by −1; Fig. 2D). As a result of this last step, positive values indicate a movement in the direction of the background motion and negative values indicate a movement in the opposite direction to background motion.

To confirm that participants consider the position of their finger when choosing between targets, we compared the finger positions 100 ms after the background started moving (before any response to the background motion) on trials in which the target in the direction of background motion or in the opposite direction was chosen. Any systematic difference between the finger positions would be evidence of the position at that moment influencing the choice between the targets. To establish that the background motion influenced how the fingers moved, we analyzed the average positions of participants’ index fingers from the onset of background motion. In particular, we analyzed the positions orthogonal to the direction from the starting point to the original target. To determine whether the response to background motion influences the choice between the targets, we assessed how often participants selected each target. Since the direction of background motion was independent of the finger’s position when the background started to move, any bias to select the target that was in the direction of background motion can be attributed to such motion. Finally, we examined whether individual biases in the choices are related to individual influences of the background. As our measure of the influence of the background, we defined the *response to background motion* as the difference between the finger’s average velocity in the direction of background motion 150 to 200 ms after the background started to move (during the first part of the response) and 50 to 100 ms after the background started to move (before the response). We defined a *choice bias* as the difference between the number of trials in which the target in the direction of background motion and in the opposite direction was chosen, divided by the total number of trials.

## Results

We refer to trials in which the finger moved to the target in the direction of background motion as *congruent* trials and trials in which it moved to the target in the opposite direction as *incongruent* trials.

### Finger position before any response to background motion

**Fig. 3:**
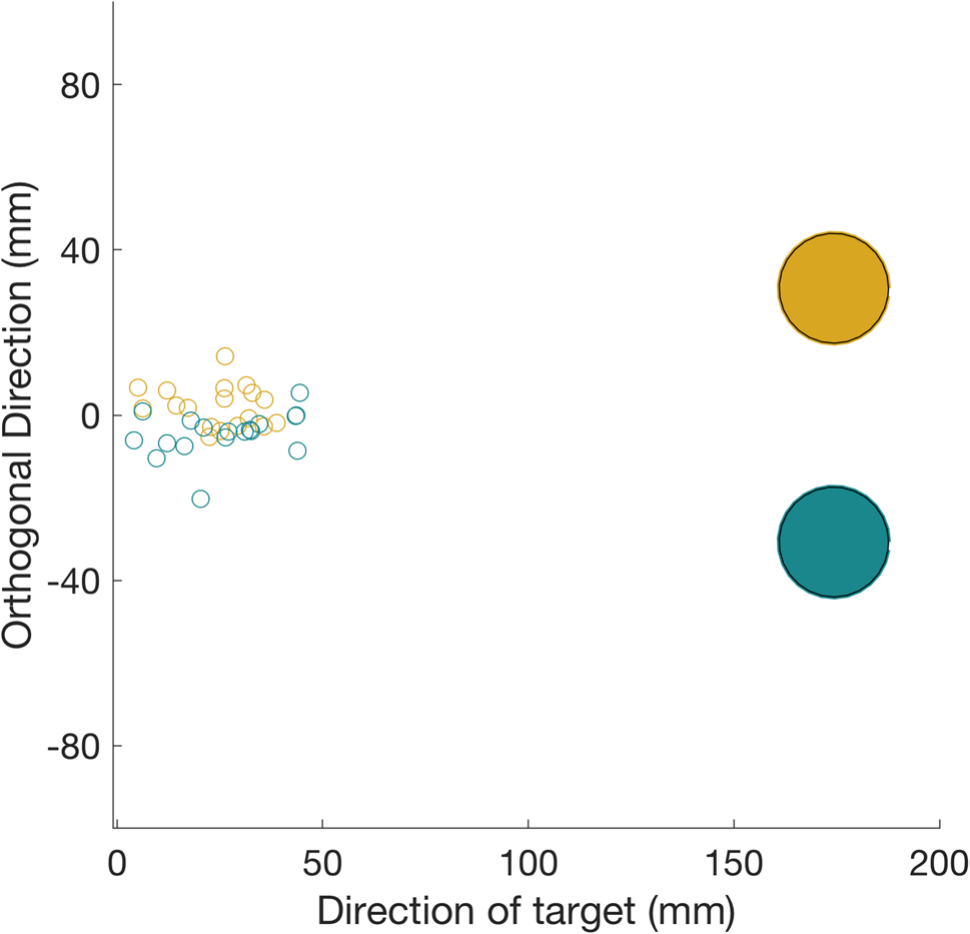
Average positions of individual participants’ fingers 100 ms after background motion onset (small circles), in congruent (gold) and incongruent (turquoise) trials. The locations of the two corresponding targets are shown to scale in the same two colours (large disks). (Colour figure online)

At 100 ms after the onset of background motion, the average finger position in the direction orthogonal to the straight path to the initial target was more positive in the congruent trials than in the incongruent trials (Fig. 3). This cannot have been a response to the background motion, because such responses only start after 120–150 ms (Crowe et al., 2021, 2022). Thus, participants were more likely to pick the target that their finger was closest to at that time (confirming earlier findings; Brenner & Smeets, 2022; Kurtzer et al., 2020).

### Finger response to background motion

**Fig. 4:**
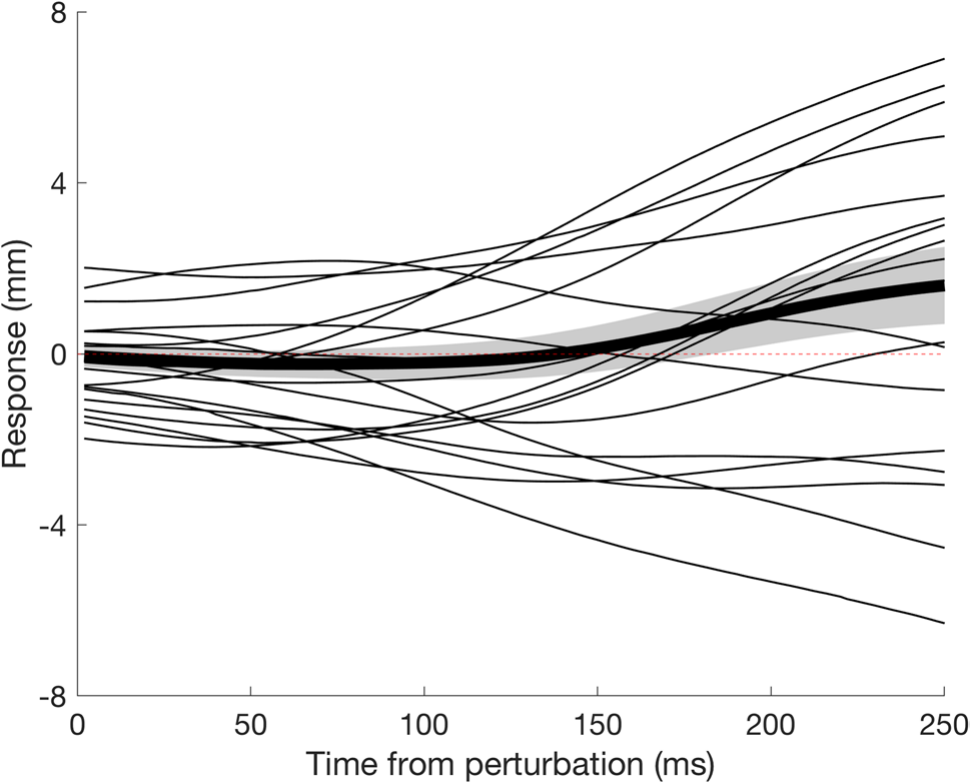
Time course of the response to background motion. The thin curves show individual participants’ mean responses. The thick curve shows the mean response across participants. The grey region around the thick curve shows the standard error of the mean across participants. A positive response is in the direction of background motion

We transformed our data such that the background was always moving upwards (Fig. 2) and refer to a change in the finger’s position in that direction as a positive response. Figure 4 shows the mean response for the first 250 ms from when the background started moving. As expected, on average the finger started following the direction of background motion about 150 ms after background motion onset.

### Target choice

**Fig. 5:**
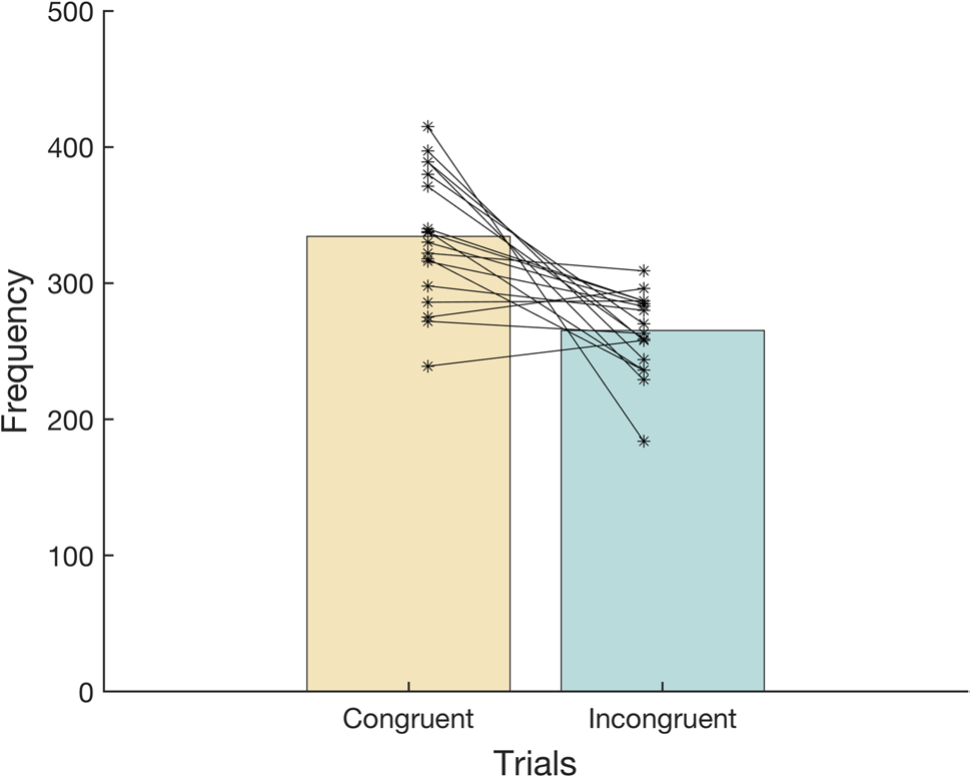
Frequencies of congruent and incongruent trials: trials in which the target in the direction of background motion and in the opposite direction were selected. Lines connect participants’ two data points. The coloured bars show the means across participants. (Colour figure online)

**Fig. 6:**
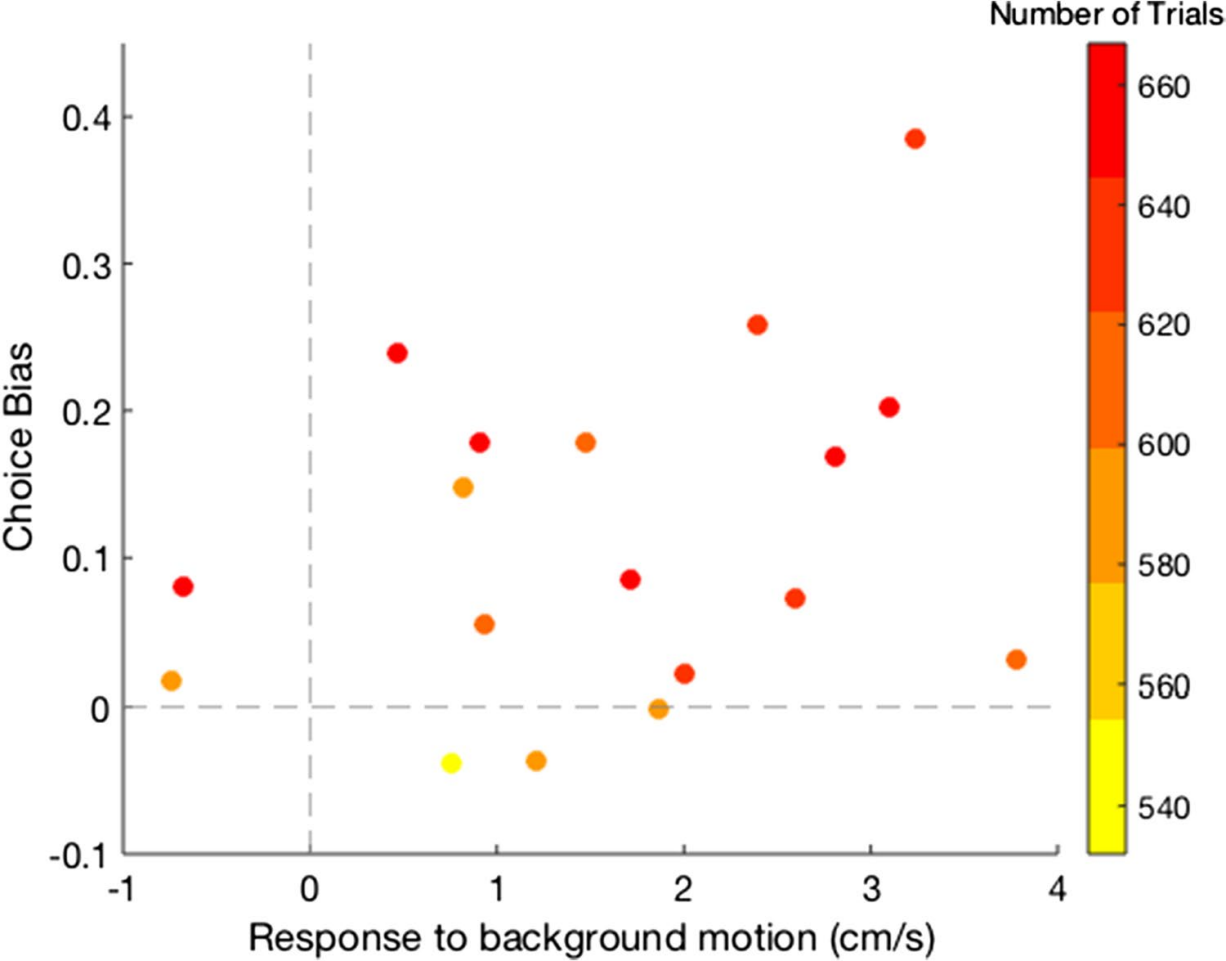
Relationship between individual participants’ responses to background motion and their choice biases. Positive values indicate that the finger’s velocity increased in the direction of background motion and that they tended to choose the target that was in the direction of background motion. The colour coding indicates the total number of trials completed by each participant. (Colour figure online)

Our main question was whether the background motion would influence participants’ choices. It did: Almost all participants chose the congruent target more frequently than the incongruent target, so there were more congruent than incongruent trials (Fig. 5). The difference between the number of congruent and incongruent trials was statistically significant: *t*(18) = 4.44 , *p* < .001 (paired *t* test), Cohen’s *d* = 1.74, 95% CI [0.97, 2.51]. Thus, the moving background enticed participants to select the target that was in the direction of background motion. Support for the idea that the (anticipated) change in the finger’s position due to the background’s motion is responsible for the choice bias can be found in the tendency for participants whose finger was shifted more by the background to also be more biased towards the congruent target: a positive correlation of 0.31 (Fig. 6). Finally, we observed that participants who completed more trials tended to have both larger responses to background motion and larger choice biases (colours in Fig. 6).

## Discussion

Having shown that participants’ choices can depend on the position of the finger (Fig. 3), that their fingers moved in the direction of background motion (Fig. 4), that they preferentially tapped on targets that were in the direction of background motion (Fig. 5), and that participants whose finger movements were influenced more by the background motion also had a larger bias (Fig. 6), it is tempting to conclude that the (anticipated) change in the finger’s position as a result of the background motion is responsible for the selection bias. Before considering alternative explanations, we will briefly consider these individual findings.

It is no surprise that the finger is pulled in the direction of motion of structures in the surrounding (Brenner & Smeets, 1997; Gomi et al., 2006; Saijo et al., 2005; Whitney et al., 2003; Zhang et al., 2018, 2019). This presumably occurs because the movement endpoint is assumed to move with such motion (Crowe et al., 2021). In accordance with this, the responses appeared to be stronger for participants who moved faster (colours in Fig. 6) and therefore had less time to adjust their movement (Brenner et al., 2022).

We know that responses to (mechanical) perturbations consider many details that are relevant to the task, such as the shape of the target and the presence of obstacles (Nashed et al., 2012), and that choices between targets can depend on the finger’s position at the moment the choice needs to be made (Brenner & Smeets, 2015b, 2022; Kurtzer et al., 2020). The aim of the current experiment was to explore whether the position (or motion) of the finger that is considered when choosing between targets is immediately updated when the movement is perturbed. We used the response to background motion to perturb the movement. Participants were more likely to choose the target in the direction of the background motion. Moreover, participants whose movements were more susceptible to background motion tended to exhibit a larger bias towards choosing the congruent target.

The results themselves are clear, but the interpretation needs some consideration. Background motion might have influenced the choice directly, or by pulling the eyes in the direction of background motion (Masson & Castet, 2002) so that the congruent target is closer to fixation. To evaluate such alternative explanations, it is useful to consider the timing of the experiment. The background started moving 200 ms after the original target appeared. The hand’s response started between 100 and 150 ms later, so 300–350 ms after the original target appeared. The eyes may have started to respond slightly earlier, although that normally only happens when the circumstances are specifically designed to induce fast responses (Masson & Castet, 2002). Thus, the background certainly did not move the hand, and probably did not move the eyes, before the choice appeared. Consequently, if information at the moment that the choices appear determines which target is chosen, it must be information about the planned eye or hand movement. This excludes the possibility that the influence of the background is caused by people choosing the target that is closest to fixation at the moment the choice appears.

We consider it unlikely that pursuing the background with their eyes influenced the decision later, because presumably participants made a saccade to the chosen target soon after the options appeared. However, eye movements were not measured to verify this. We cannot be sure whether the background’s influence on the *planned* movement at the moment the choice appeared (which obviously preceded the actual movement) or its influence on the actual movement some time later, influenced the choice. To find out we would have to determine the time window during which choices are influenced by moving the background. What we have established is that very recent information about one’s posture is considered when choosing between options.
